# Early Identification of Rotten Potatoes Using an Electronic Nose Based on Feature Discretization and Ensemble Convolutional Neural Network

**DOI:** 10.3390/s24103105

**Published:** 2024-05-14

**Authors:** Haonan Lin, Zhenbo Wei, Changqing Chen, Yun Huang, Jianxi Zhu

**Affiliations:** 1 Department of Biosystems Engineering, Zhejiang University, 866 Yuhangtang Road, Hangzhou 310058, China; 22113001@zju.edu.cn; 2 Zhejiang Academic of Agricultural Machinery, 1158 Zhihe Road, Jinhua 321051, China; jhnjsccq@163.com (C.C.); cobra21cn@163.com (Y.H.); zhujx2015@163.com (J.Z.)

**Keywords:** rotten potato, electronic nose, ensemble neural network, discrete analysis, pattern recognition methods

## Abstract

The early identification of rotten potatoes is one of the most important challenges in a storage facility because of the inconspicuous symptoms of rot, the high density of storage, and environmental factors (such as temperature, humidity, and ambient gases). An electronic nose system based on an ensemble convolutional neural network (ECNN, a powerful feature extraction method) was developed to detect potatoes with different degrees of rot. Three types of potatoes were detected: normal samples, slightly rotten samples, and totally rotten samples. A feature discretization method was proposed to optimize the impact of ambient gases on electronic nose signals by eliminating redundant information from the features. The ECNN based on original features presented good results for the prediction of rotten potatoes in both laboratory and storage environments, and the accuracy of the prediction results was 94.70% and 90.76%, respectively. Moreover, the application of the feature discretization method significantly improved the prediction results, and the accuracy of prediction results improved by 1.59% and 3.73%, respectively. Above all, the electronic nose system performed well in the identification of three types of potatoes by using the ECNN, and the proposed feature discretization method was helpful in reducing the interference of ambient gases.

## 1. Introduction

Potatoes are the fourth most widely cultivated crop in the world because of their rich starch, protein, and nutrient content [1,2]. The global potato production has surpassed 376.1 million tons and more than 70 countries have listed potatoes as their staple food (Food and Agriculture Organization, 2021). China is the largest potato producer, and its potato yield and planting area account for over 25% and 32% of the world’s total production (China National Bureau of Statistics, 2022). However, the post-production loss of potatoes can reach up to 20–25% each year due to improper storage and diseases [3]. Latent diseases flourish under improper storage conditions, causing tissue damage, internal fermentation, and bacterial or fungal infection. As the degree of rot increases, the degradation of lipid substances in the potato produces more aldehyde components [4], such as malondialdehyde [5]. Infection increases the content of soluble proteins, which promote the production of ketones and aldehydes, such as octene, a-phellandrene, cyclohexanone, heptaldehyde, acetaldehyde, etc. [6]. Furthermore, starch also decomposes into glucose, which can induce the Maillard reaction with amino compounds, and the consumption of starch leads to the appearance of furan and pyrazine [7,8,9]. During the rotting process, potatoes produce more specific volatile components, which can be used for the detection of rotten potatoes in the early stages.

In the past, the traditional method for detecting rotten potatoes was manual inspection, which is labor-intensive and inefficient. Recently, there has been a lot of research focusing on identifying rotten potatoes automatically, including using computer vision [10,11,12], spectral technology [13,14], GC-MS [15,16], and GC-IMS [17,18]. However, rotten potatoes are always hidden in piles when potatoes are stacked together. Computer vision can only detect surface changes and spectral energy is insufficient to explore internal alterations. Although the GC-MS/GC-IMS method can be applied to detect rotten potatoes inside piles based on volatile components, it is hard to achieve online detection due to the unavailability of professional gas collection devices and the complex pre-treatment process. 

The electronic nose (E-nose), an odor-based, rapid, portable, and sensitive technology [19], has been introduced as a promising solution for investigating the rotting process in different agricultural products. Wang et al. [20] pointed out that rotten kiwifruit can produce lipids, alcohols, and aromatic hydrocarbons through metabolic processes which change the composition of volatile compounds in kiwifruit, and that there is a close correlation between E-nose signals and metabolites (*p* < 0.01). Wang et al. divided rotting kiwifruit into latent, early, middle, and late stages based on different storage times, and kiwifruit in the latent stage of rot could not be recognized [21]. Liu et al. reported that microbial numbers in strawberries inoculated with B. cinerea spores could be effectively and quantitatively predicted at different time intervals based on a fusion dataset using the E-nose and hyperspectral data (R^2^ = 0.925, RMSE = 0.38) [22]. Wijaya et al. used the E-nose to monitor the freshness levels of beef and classified beef into different quality levels using the E-nose (the accuracy, respectively, reached 93.64%, 93.64%, and 86%) [23]. Overall, the E-nose has been proved to be a potential tool to identify different quality levels of agricultural products based on volatile compounds. However, these studies have only been validated in the lab environment, and the experiments were characterized by limited numbers samples and standardized containers, therefore rarely providing a detection mode that would be suitable for use in actual storage facilities.

Some studies have used electronic noses for the early detection of rotting potatoes. Zhiyong Chang et al. predicted potatoes with different decay proportions in the laboratory and the average accuracy was up to 83.8% [24]. E. Biondi et al. used a commercial electronic nose to detect potatoes with different storage scales and completed a visualization of normal potatoes and diseased potatoes through PCA [25]. Rutolo, M.F. et al. reported the effectiveness of the electronic nose in detecting potato soft rot in the laboratory using machine learning methods such as LDA, PCA, SVM, etc. [26]. They placed a newly developed gas analysis device composed of gas sensors near rotting potatoes which were stored together and separated from normal potatoes. Thanks to periodically recorded signals of sensors, they artificially estimated the recognition threshold of rotten potatoes [27]. Ghosh, A. et al. designed a VOC detection device called e-POT to periodically detect the VOCs of potatoes in cold storage after verifying the existence of a decay threshold in potatoes [28]. These studies have shown promising results but rarely considered the impact of ambient gases, especially those in a humid environment with lots of agricultural products. However, complex ambient gases will obviously affect the detection of rotten potatoes during the long-term storage phase, which is one of the reasons why the E-nose is unlikely to achieve online detection in realistic scenarios.

In this study, an E-nose based on an ensemble convolutional neural network (ECNN) was developed for the detection of rotting potatoes in storage, and a feature discretization method was used to reduce the interference of ambient gases. The aims of this study were (1) to develop a portable E-nose for the detection of rotten potatoes; (2) to evaluate the effectiveness of an E-nose based on an ECNN and a feature discretization method for the identification of rotten potatoes.

## 2. Materials and Methods

### 2.1. Sample Preparation

**Figure 1:**
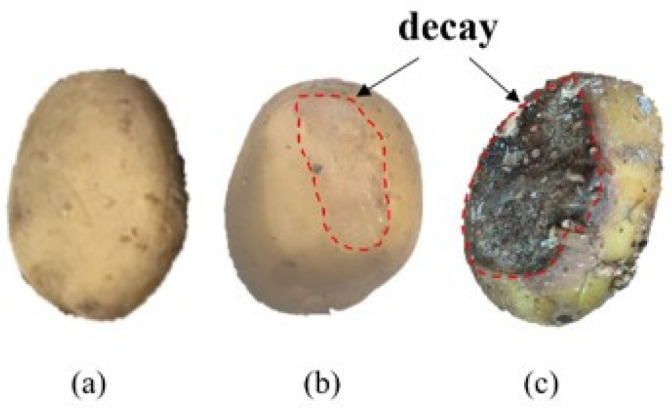
Appearance of normal potatoes and rotten potatoes: (**a**) normal potato, (**b**) slightly rotten potato, (**c**) totally rotten potato.

Potato tubers of the variety Zhongshu No.5 were harvested from Jinhua Academy of Agricultural Sciences (Jinhua City, Zhejiang Province, China) in November 2022. After being stored in a cool and dark environment (15 °C ± 0.5 °C) for two weeks in order to dry and to allow for the distinction of diseased specimens, the potatoes were stored in a ventilated storage facility (4–8 °C, 85–90% RH) for 4 months. One hundred each of normal potatoes, slightly rotten potatoes, and totally rotten potatoes were randomly selected based on symptoms of rot. Slightly rotten potatoes were characterized by soft tissue, spots, and a slight odor [29], and totally rotten potatoes usually had local damage and a visible bacterial colony on the periderm. The internal tissues of the potatoes gradually transformed into a sponge-like or viscous fluid consistency and finally became black because of fermentation and oxidation while emitting a pungent odor [30]. The appearance of the different categories of potatoes is shown in Figure 1. During the test, these potatoes were mixed with normal potatoes to produce a sample weighing 10 kg in total. Overall, a total of 300 potato samples were prepared, which could be divided equally into three categories (normal sample, slightly rotten sample, totally rotten sample).

### 2.2. The Electronic Nose Detection System

**Figure 2:**
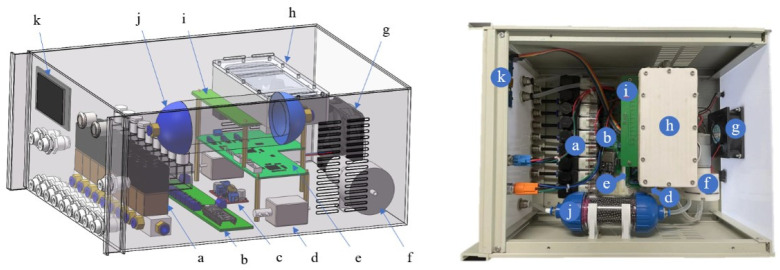
Schematic diagram of the E-nose. The components of the E-nose are as follows: (a) 9-channel solenoid valve, (b) channel control module, (c) power module, (d) solenoid valves, (e) MPU board, (f) air pump, (g) cooling fan, (h) gas chamber, (i) sensor array sample circuit board, (j) air filter (activated carbon and Cupric sulfate), (k) display screen.

**Figure 3:**
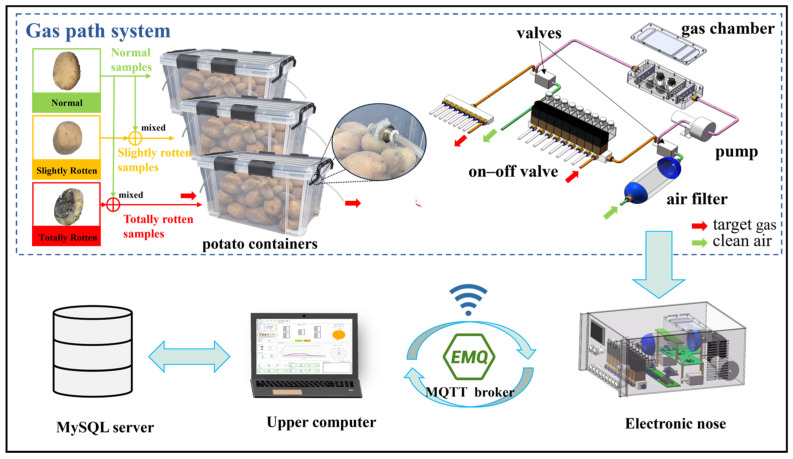
Schematic diagram of E-nose system.

The electronic nose system includes servers that support MySQL and MQTT, an electronic nose, and an upper computer with a self-designed visualization software. The E-nose mainly consists of a main processor unit (MPU) board, a circuit board for sampling, and a channel control module. A schematic diagram of the E-nose is shown in Figure 2. The visualization software in the upper computer is used for wireless transmission, signals visualization, data storage, and pattern recognition. The whole detection system is shown in Figure 3.

The main processor is able to receive orders from the upper computer by Wi-Fi to control the gas path, implement the detection function, and send data to the upper computer client software. The gas path module is used to pump clean air or the target gas into the gas chamber. The channel control module can control 9 channels to collect gas. The voltage signals of 12 semiconductor metal oxide sensors are collected by the sensor array board and converted from an analog quantity to a digital quantity.

The gas path system consists of a miniature vacuum pump, Teflon tubes, an activated carbon filtration device, two two-position three-way valves, a sensor chamber, and a 9-position on–off valve. Storage boxes (about 20 L) were selected to store the samples and collect volatile gases. The 9-position on–off valve allows the E-nose to automatically and periodically monitor the quality of potatoes in multiple storage boxes. The sensor chamber is made of Teflon, and it can provide the sensor arrays with an enclosed space for making sufficient contact with target gas. The whole E-nose system is shown in Figure 3.

A past study reported that the volatile compounds of rotten potatoes mainly include aldehydes, ketones, esters, amines, alcohols, olefins, ethers, and furans [5], and this information on volatile compounds was used for E-nose sensor selection.

**Figure 4:**
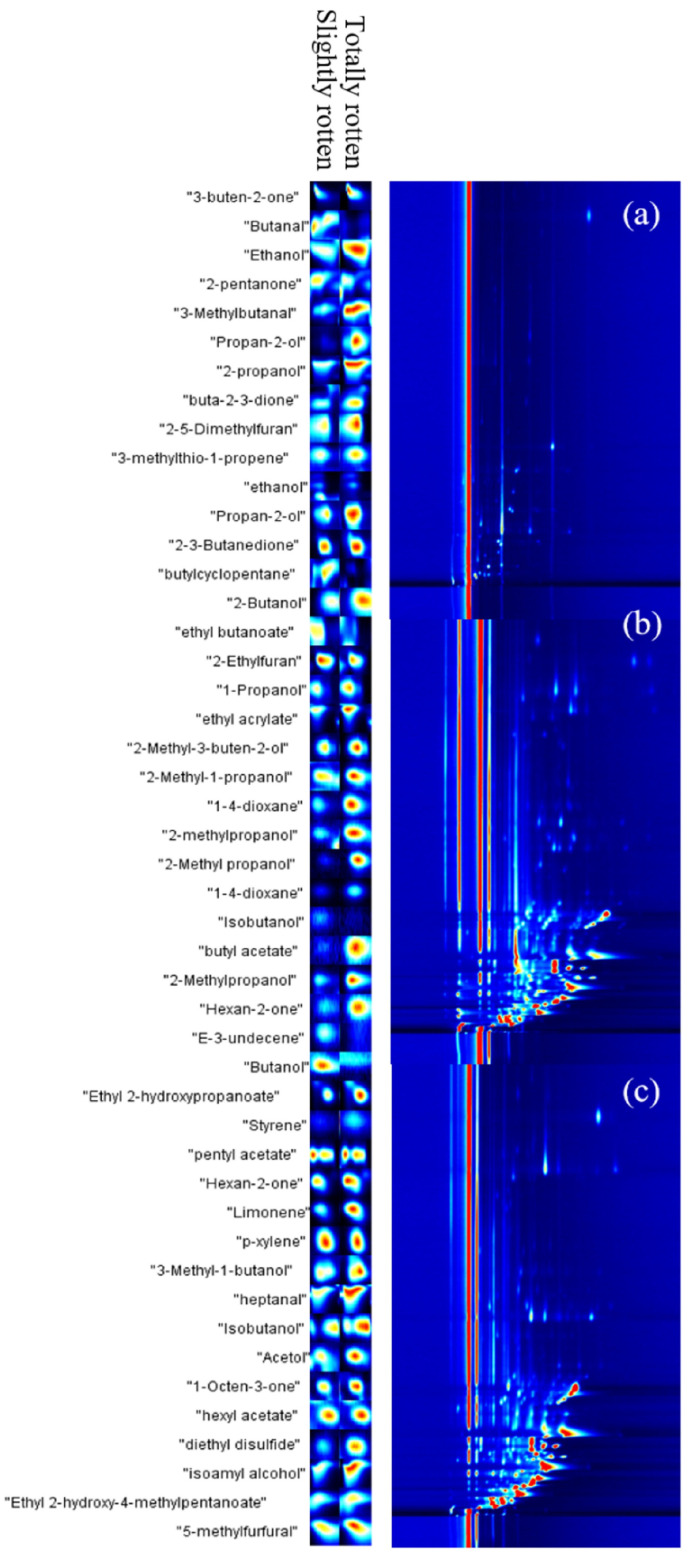
GC-IMS results of normal, slightly rotten, and totally rotten potatoes: (**a**) normal potatoes; (**b**) slightly rotten potatoes; (**c**) totally rotten potatoes.

We collected 10 g samples of normal, slightly rotten, and totally rotten potatoes and put them into 20 mL bottles. After 10 min of headspace, gases were analyzed using GC-IMS, and the results are shown in Figure 4. The closer the color is to red, the higher the concentration of specific compound. The closer the color is to blue, the lower the concentration. From Figure 4, it can be seen that there are differences in basic VOCs between slightly rotten potatoes and totally rotten potatoes. The volatile components emitted from rotten potatoes include 4 types of aldehydes, 9 types of ketones, 12 types of alcohols, 6 types of esters, 6 types of alkenes, 2 types of furans, 2 types of aromatic hydrocarbons, and 1 type of sulfide. Some representative components of these VOCs, such as glyoxal, heptanal, styrene, isobutanol, sulfide compounds, and furan compounds, were also reported in another study [5].

Popular gas sensors include metal oxide semiconductors (MOSs), electrochemical, infrared, and catalytic combustion sensors [31]. Catalytic combustion sensors mainly target combustible gases and have poor selectivity. Infrared sensors are usually expensive and are easily affected by temperature and humidity. Electrochemical sensors have long response times, especially in low-temperature environments where the reaction rate is slow, making it difficult to fully represent real-time signal changes during gas adsorption and desorption processes [32]. MOS sensors are the most mature and cost-effective, characterized by diverse categories, good real-time properties, and good stability [33].

According to the results of GC-IMS and sensor characteristics, 12 MOS sensors were selected to build a sensor array, and the characteristics of these sensors are listed in Table 1.

### 2.3. The Detection Procedure of the E-Nose

Experiments with the same procedures were conducted in both the laboratory and the storage facility. The experimental parameters (Table 2) were trialed many times in pre-experiments on the basis of previous studies conducted by our team [34,35,36]. Specifically, headspace time depended on the peak values of sensors at different times. Pre-heating time was determined when the response signals of all sensors reached a stable state. Pre-cleaning time verified the real-time resistance of each sensor in the visualization software until there was no change to any of the resistances by continuously intaking clean air for a period of time. After the pre-cleaning time, the sensors were no longer affected by the temperature and humidity of input gases, and the response signals could be initialized to a baseline. The criteria of injection time and cleaning time were that half of the sensors reached a stable response value or decreased to baseline (±0.3).

The detection process was as follows: (1) the storage box containing the sample was kept for 10 min to generate the headspace gas; (2) Teflon tubes were used to connect the box with the E-nose so that the headspace gas could be pumped into the sensor chamber; (3) clean air filtered by activated carbon particles was pumped into the gas chamber for 60 s to initialize the sensors to base values; (4) the headspace gas of the sample was then pumped into the gas chamber to produce response signals for 90 s till the response signal had no changes; (5) the signals of the sensors reached the base values during the filtering stage and clean gas washed the sensor array for 90 s. A total of 12 (number of sensors) × 180 (response values) = 2160 data items could be collected for each sample.

Data were individually collected and detected in the lab and in the storage facility. All data were marked as D ∈ R^600×12×180^ and contained data detected in the laboratory, D1 ∈ R^300×12×180^, and data detected in the storage facility, D2 ∈ R^300×12×180^.

### 2.4. Data Preprocessing

A relative difference method based on the conductivities of the sensors in clean air was used to correct the baselines. The sensors’ long-term drift that occurs in clean air would partially offset the drift in target gases by using the relative differential conductivities as response signals. It is helpful to reduce the impact of long-term drift on the sensors of the electronic nose. The response values of the sensors were normalized using the following equation:

(1) $$x^{\left(i\right)}=\frac{R_{air}^{\left(i\right)}}{R_{S}^{\left(i\right)}}=\frac{G^{\left(i\right)}}{G_{0}^{\left(i\right)}}$$

where $x^{\left(i\right)}$ is the response of the i-th sensor, $R_{air}^{\left(i\right)}$, $G_{0}^{\left(i\right)}$ are the average resistance and conductivity of the i-th sensor in clean air, and $R_{S}^{\left(i\right)}$, $G^{\left(i\right)}$ are the real-time resistance and conductivity of the i-th sensor in the sample gas. 

The response signals of the E-nose are a kind of high-dimensional data and would easily encounter the problem of curse of dimensionality during data processing. In order to catch crucial information from response signals to provide assistance for pattern recognition, it is necessary to extract the low-dimensional representation of row data. Based on previous studies [29,30,31] conducted by our team and on the characteristics of the developed E-nose, specific feature methods used in the experiment are shown in Table 3.

These features values are normalized to the range [0, 1] by the following normalization equation:

(2) $$F_{\mathrm{normal}}=\frac{F_{\mathrm{original}}-F_{\min}}{F_{\max}-F_{\min}}$$

where $F_{\mathrm{original}}$, $F_{\max}$, $F_{\min}$ are the original features, the maximum feature value, and the minimum feature value, respectively. The data of each sample contain 12 (number of sensors) × 12 (feature value) values after data preprocessing.

To grasp the meaningful difference between different samples, we used the MIME-(SVM-RFECV) method [34], which is a kind of feature selection algorithm proposed in a previous study of our research team to screen for correlative features and remove irrelevant redundancy. This method includes two steps: filter and wrapper. In the filter stage, mixed mutual information between each feature and the rest is calculated by Equation (3) and features below the specific threshold are filtered. An iterative strategy is introduced in the wrapper stage; we randomly remove a previously unselected feature from the feature dataset of the previous iteration and train the SVM model with the rest of the features. Comparing the new result with the previous result, we could judge the importance of the deleted feature and decide whether to delete this feature. By repeating the above process, the optimal feature dataset would be determined.

(3) $$\mathrm{MIME}(F_{i})=I(F_{i},y)-\alpha \frac{1}{m}\sum_{j=i}^{m}I(F_{i},F_{j})-\beta \frac{1}{m}\sum_{j=i}^{m}I(F_{i},F_{j}|y)$$

**Figure 5:**
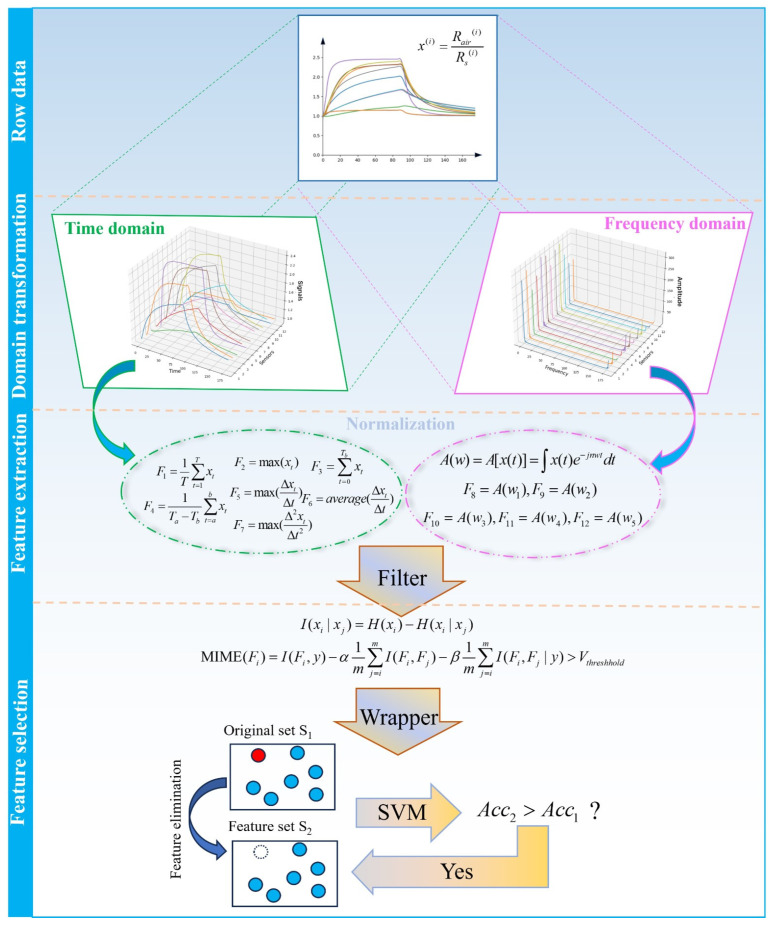
The flow of data preprocessing for E-nose data. The red circle indicates that the selected feature in the original set; The blank circle indicates that the feature is deleted in the elimination process; The blue circles represent other features that are not selected in this iteration.

Eventually, 29 features were selected from the original features to produce the final set of features. The whole flow of data preprocessing is shown in Figure 5. Finally, the feature dataset of two experiments could be denoted as F1, F2∈R^300×29^. Each dataset F is randomly divided into a training dataset F_train_∈R^180×29^, a test dataset F_test_∈R^90×29^, and a validation dataset F_val_∈R^30×29^.

### 2.5. Electronic Nose Data Analysis Methods

In a storage environment, there are often complex interference gases because of poor ventilation, damp, and a lack of regular cleaning. These ambient gases, which are irrelevant to the sample categories, would affect the response signals and increase redundant information in data features. In order to solve the problem of information interference from ambient gases, we proposed a feature discretization method that differentially labels the redundant information of features and helps the classification model better focus on significant differences. Once more, we built an ensemble convolutional neural network which was good at multi-scale feature extraction and distribution fitting by using multiple different CNNs and ensemble learning to reduce the influence of interference and improve the stability and accuracy of our electronic nose.

#### 2.5.1. Ensemble Convolutional Neural Network

The ensemble convolutional neural network consists of two parts (base classifiers and an ensemble CNN). The first part is composed of 10 base classifiers, which are based on one-dimensional convolutional neural networks (CNNs) with different convolutional kernel sizes and hyperparameters. The second part (ensemble CNN) is a single convolutional neural network which makes ensemble decisions based on the outputs of the first part. The activation functions of all layers are ReLU.

The base CNN consists of two convolutional layers and two multilayer perceptron (MLP) layers. The parameters are initialized using Gaussian distribution. For the first layer, the range of convolutional kernel sizes is [3, 5, 7, 9] and the range of kernel numbers is [8, 16, 32, 64]. And for the second layer, the ranges of kernel sizes and numbers are [3, 5, 7] and [32, 64, 128]. The strides of two convolutional layers are both 2. The sizes of the MLP layers are (n, 200) and (200, 3), where n indicates the size of the convolutional layer’s output after a “concatenate” operation. Thus, the number of hyperparameter groups is up to 144. The datasets F_train_ and F_val_ were used to train and validate each CNN model separately (learning rate = 0.001, decay = 0.9, epoch = 300) and the prediction accuracy was regarded as the evaluation standard. The optimal CNN and the corresponding hyperparameter groups, which are (5, 64) and (3, 128), were determined by the grid search method. In order to obtain different CNNs as other base classifiers, we changed the sizes and numbers of kernels in given ranges centered around the optimal group. Finally, 10 different CNNs with different hyperparameter groups were selected as the base classifiers to provide a multi-scale feature extraction.

**Figure 6:**
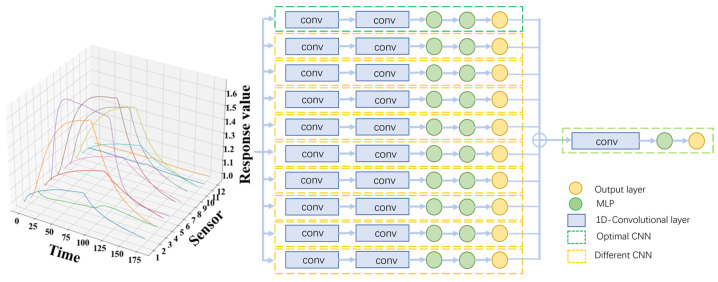
Diagram of the ensemble convolutional neural network. The optimal CNN has the highest prediction accuracy in all parameter groups. Different CNNs are obtained by changing the parameters based on the optimal CNN.

The ensemble CNN was utilized to make an ensemble decision by automatically learning the weights of different base classifiers. The CNN includes 1 convolutional layer and 1 MLP layer. The range of kernel sizes is [3, 5, 7], the range of kernel numbers is [4, 8, 16, 32, 64]. After the base classifiers were trained, the results of the base classifiers were fed into the CNN and the CNN output the final prediction result. The optimal hyperparameter groups of the CNN, which are (3, 16), were also determined with the same process. The whole model structure is shown in Figure 6.

#### 2.5.2. Feature Discretization Method

Affected by environmental odors and by the similarity of sample odors, an overlap of feature value ranges between different sample categories is often present in E-nose data. This can be interpreted as the influence of redundancy and is irrelevant to differences between the categories. Therefore, we proposed a feature discretization method that improves on the equal-width discretization method for E-nose data to dynamically divide feature ranges based on sample categories. 

**Figure 7:**
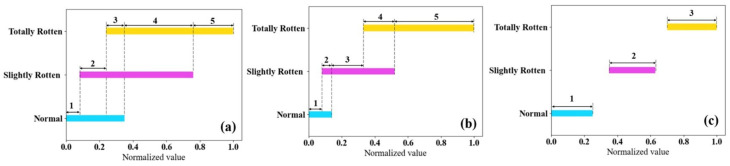
Discretization analysis of feature discretization: (**a**) high overlap; (**b**) partial overlap; (**c**) no overlap.

Maximum and minimum values of each feature for all samples were calculated individually. The ranges of features were divided into several segments depending on the overlap of feature values. Features within the same segment were mapped to a new tagged value, such as 1, 2, 3, etc. The re-marked features were transformed into the form of one-hot vectors with a value of one, whose dimension represents the corresponding tagged value after discretization. A sketch map of feature discretization is shown in Figure 7. Figure 7a–c successively present the situations of high overlap, partial overlap, and no overlap.

## 3. Results

### 3.1. The Response Curves Obtained by the E-Nose

**Figure 8:**
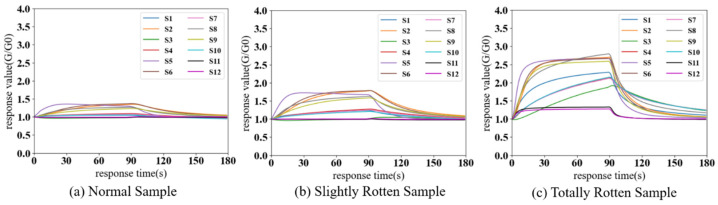
Response curves of different categories of potato samples detected by the electronic nose in the laboratory. The X-axis represents the response time of the electronic nose; the Y-axis represents the response values of sensors.

Figure 8 shows the response value curves of different categories of potato samples detected by the electronic nose in a laboratory environment. As can be seen in Figure 8, the response intensity to the totally rotten sample is the largest, while that of the normal sample is the smallest. According to Table 1, sensors S1, S4, S8, and S10 have selectivity for hydrogen, aromatic hydrocarbons, alkanes, ketones, ethanol, and formaldehyde. Their response signals to normal samples are weak, but there are significant increases in response to slightly rotten and totally rotten samples (the peak values of S4 in normal, slightly rotten, and totally rotten samples are 1.11, 1.27, and 2.05, respectively). S2, S5, and S9 are all highly sensitive to ethanol or other alcohols. The response signals of these sensors to the three categories of samples are obvious and show an increasing trend as the rot process of potatoes progresses (the peak values of S5 are 1.39, 1.73, and 2.67). Sensors S11 and S12 are sensitive to alkanes, and only totally rotten samples produce a relatively high response due to the high detection limits of these two sensors. Sensor S3 is sensitive to ammonia and hydrogen sulfide and a high response value only appears for totally rotten potatoes. To sum up, rotten potatoes mainly volatilize aromatic hydrocarbons, alkanes, alcohols, ketones, and aldehydes, and the concentration of these gases will increase as the degree of rot deepens. In particular, in totally rotten potatoes, internal fermentation may produce hydrogen, hydrogen sulfide, and ammonia.

**Figure 9:**
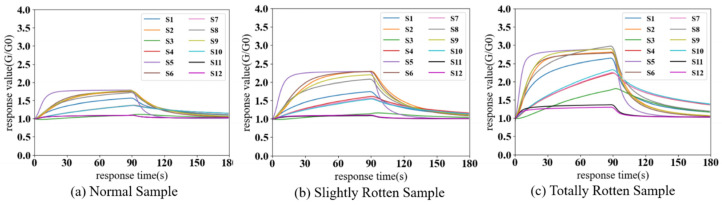
Response curves of different categories of potato samples detected by the electronic nose in storage. The X-axis represents response time of the electronic nose; Y-axis represents the response values of sensors.

Figure 9 shows the response curves of different categories of samples detected by the electronic nose in the storage facility. Variations in sensor peak values are easily observed across different categories of samples, exemplified by the response values of S8, which are 1.71, 2.01, and 3.03, respectively. The response signals of S1–S10 to normal and slightly rotten samples clearly increased, as seen in Figure 9, which might be due to the presence of complex ambient gases in storage. The response signals to totally rotten samples show relatively limited changes because of the high concentration of volatile compounds produced by totally rotten potatoes and to the cross-sensitivity and nonlinear response characteristics of MOS sensors. But there are still visible response increases in some of the sensors, such as S1, S5, S8, and S9. Therefore, ambient gases can affect the E-nose by increasing the response signals and make it difficult to identify rotten potatoes or distinguish between different degrees of rot.

### 3.2. Visual Analysis of Data in Laboratory and Storage Environments

**Figure 10:**
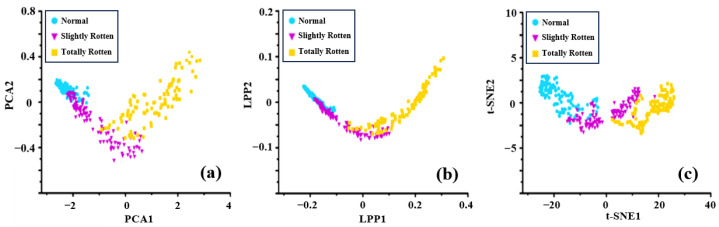
Visualization results of different categories of potato samples detected by the electronic nose in the laboratory: (**a**) PCA; (**b**) LPP; (**c**) t-SNE.

**Figure 11:**
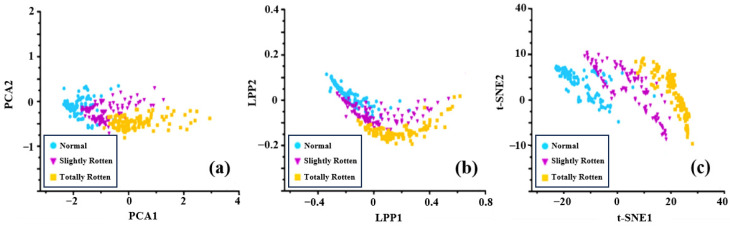
Data visualization results of different categories of potato samples detected by the electronic nose in storage: (**a**) PCA; (**b**) LPP; (**c**) t-SNE.

Dimension reduction algorithms (t-SNE [37], PCA [38], and LPP [39]) were used to analyze the feature dataset, followed by feature selection. The analysis results are shown in Figure 10 and Figure 11.

Figure 10 shows the data visualization results of different categories of potatoes detected by the electronic nose in the laboratory. The points of normal samples are the most concentrated because of the similarity of normal potatoes’ odors from the perspective of the dispersion degree of data points. In Figure 10a,b, the points of slightly rotten samples partially overlap with those of normal samples and totally rotten samples. The points of slightly rotten samples and totally rotten samples are more scattered than those of normal samples, which can be interpreted as differences in the volatile components produced by rotten potatoes under the same storage time. The samples of the three categories could not be easily distinguished in the results of LPP and PCA in a two-dimensional space, which was possibly because of key differential information lost during the dimensional reduction process. The t-SNE method performed relatively better than others by preserving the spatial distribution of original features.

Figure 11 shows the data visualization results of different categories of samples detected by the electronic nose in a potato storage space. It is obvious that the introduction of ambient gases led to a more dispersed trend in the low-dimension space due to the influence of noise information and the interference of complex gases, especially for normal samples. The data points are more clustered and the distributions of samples from different categories overlap with each other because ambient gases increase redundant information and mask the differences between the categories (Figure 11a,b). The t-SNE method also presented a poor effectiveness in distinguishing samples of different categories because ambient gases affected the raw signals and the distribution of features (Figure 11c). Therefore, it is necessary to increase the differences between different categories of features and reduce the impact of redundant information on the prediction model.

**Figure 12:**
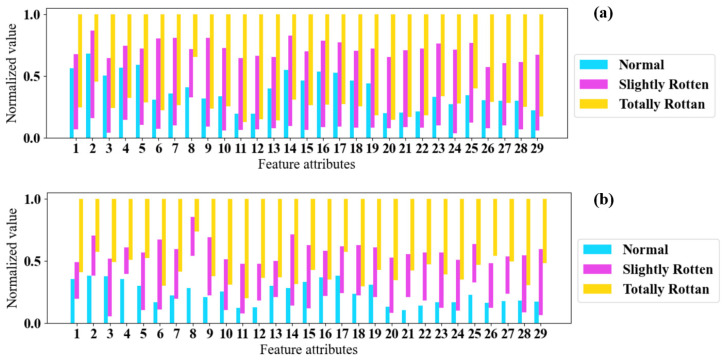
Maximum values and minimum values of feature attributes: (**a**) in storage; (**b**) in the laboratory.

Figure 12 shows the maximum and minimum values of the feature attributes obtained in the laboratory and in storage. The 29 features collected in the laboratory have fewer overlapping segments, which means ambient gases in storage (the smells of surrounding rotten potatoes, other agricultural products, and musty metal brackets) brought redundancy to the E-nose’s data and made features of samples from different categories hard to distinguish. This conclusion is consistent with the results of feature visualization.

### 3.3. Classification Result Analysis Based on the Feature Discretization Method

**Figure 13:**
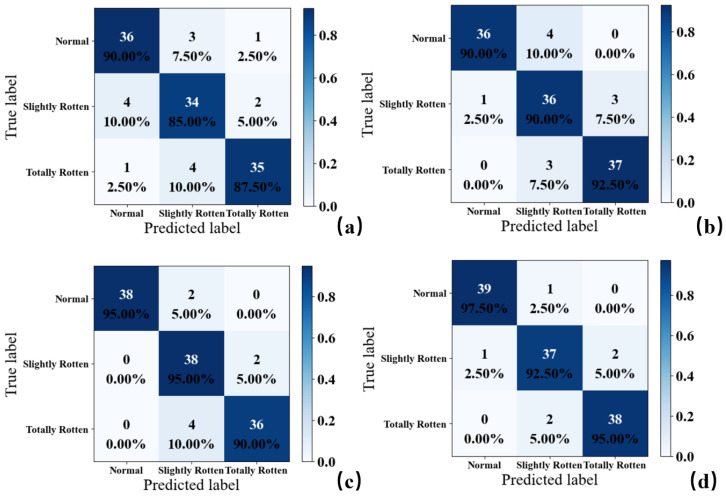
Confusion matrixes of ensemble convolutional neural network with different conditions: (**a**) storage, without discretization; (**b**) storage, discretization; (**c**) laboratory, without discretization; (**d**) laboratory, discretization.

F_train-1_ and F_train-2_, respectively, denote the training dataset of F1 and F2, and other datasets are also marked in this way. The training datasets F_train-1_ and F_train-2_, respectively, were fed into several popular classification methods, such as SVM, LR, KNN, CNN, and ECNN, to predict the degree of rot in potatoes. The prediction effects of these models were judged by the accuracy of F_test-1_ and F_test-2_. Furthermore, the feature discretization method was used to process the dataset. Each model was trained five times and information on the models’ accuracies was recorded, and the final results are shown in Table 4 and Table 5. The confusion matrixes of the model we proposed with different conditions are shown in Figure 13.

In Table 4, it is easy to find that the prediction accuracies of all methods on the test dataset were more than 84% and the prediction result of the ECNN was the best (93.11%). The result of the ECNN model was 2.12%, which was higher than that of the single-CNN model. This proves that an ensemble structure successfully increased the prediction ability of the model. Furthermore, most of the models trained by discretized features had higher accuracies, except for the SVM model, which might be because the transfer from discretized features to a high-dimensional space via a kernel function prevented the SVM model from finding a better demarcation of samples with different categories. The KNN had the biggest improvement (2.80%) and the ECNN model achieved the best classification performance (94.70%) in the laboratory. The KNN model obtained the best improvement because the process of discretization changed the distribution of feature points by unifying different feature values into the designed tags.

Table 5 shows that in the storage facility, the ECNN still had the highest test accuracy, up to 87.03%, which was 9.08%, 4.98%, and 16.2% higher than that of the SVM, LR, and KNN methods, respectively, and 2.74% higher than that of the single base classifier. However, the accuracies of all models declined compared to the laboratory experiment, especially for KNN, SVM, and CNN. The performance of KNN had a notable drop when potatoes were detected in storage, which might be because the scattered and overlapping distribution of data in the feature space was difficult for a distance-based method such as KNN. The results show that complex ambient gases had a significant interference in the detection of the degree of rot in potatoes in a storage environment because ambient gases brought irrelevant redundancy which increased the overlap area of features in the different categories and hid the differences of original feature distribution. 

When using the feature discretization method, all models had better classification performance on the test dataset and the prediction accuracy of ECNN was the highest (90.76%). The accuracy of the KNN model also had the most significant increase (8.56%) after discretization. The reason might be that the feature discretization method enlarged the differential information of features by re-marking them with different numbers and reduced redundant information by mapping feature values in overlapping areas to the same values. This process would have made the classifier more sensitive to the differences between categories of features.

Overall, the fact that the gases in storage were more complex than those in the laboratory might have resulted in a worse result of the electronic nose on the same test dataset in the storage facility. The feature discretization method effectively improved the accuracy of models in this study and reduced the impact of environmental odors on detection results. The ensemble convolutional neural network had a better classification accuracy and had the ability to resist the interference of external gases in prediction compared with other popular classification algorithms.

## 4. Discussion

According to statistical data from the National Bureau of Statistics, in 2022, China’s potato planting area reached 4.558 million hectares, of which 18.51 million tons potatoes were produced, with an average yield of about 4050.5 kg per hectare. In 2022, the export volume of potatoes was 451,800 tons, with an export value of USD 248.75 million. However, rotten potatoes cause annual losses of up to 15–25%, resulting in billions of dollars in economic loss. 

Previous studies rarely considered the impact of ambient odors on practical applications of E-noses, especially in potato storage facilities. The aim of this study was to deal with the problem of low detection accuracy of rotten potatoes in storage by E-nose because of ambient gases. The feature discretization method and the ECNN model presented excellent performance in the early detection of rotten potatoes and made the online detection of rotten potatoes possible despite the interference of ambient gases. Compared to the detection of small amounts of samples in laboratories, this detection mode of rotten potatoes in storage can effectively reduce the search scope and workload and build a foundation for subsequent precise inspections. Storage managers can quickly locate the storage units containing rotten potatoes, inspect the potatoes inside them through manual searches, machine vision, or other methods, and precisely remove rotten potatoes.

However, storage spaces would need to be equipped with multiple E-noses to effectively monitor rotten potatoes, and manual inspection is still required to remove every rotten potato after the detection of rotten potatoes in batch. These reasons hinder the success of commercializing the E-nose developed here and are also a problem that needs to be addressed in subsequent research.

### 4.1. Effectiveness of the Feature Discretization Method

Many studies have pointed out that appropriate discretization can improve the performance of classifiers [40,41], while inappropriate discretization methods lead to a reduction in classification accuracy and robustness [42,43]. Rajbahadur reported that discretization thresholds based on statistical information for continuous variables would introduce discrete noise, which would have a negative impact on the performance of classifiers due to the high complexity of noisy points around the discretization threshold [44]. Esme [45] studied the impact of Ayyad and Irani’s [46] discretization method on the detection accuracy of different classification models and validated them on a publicly available gas [47] dataset. He noted that the discretization process reduced the complexity of sensor signals and the potential impact of outliers on the classifier. 

The feature discretization method we proposed is a kind of supervised discretization, which is based on differences in class information rather than statistical information on overall samples. The distribution of noise points around the discrete threshold represents the critical state of different categories, which is meaningful for classifiers. The point is that our feature discretization method focuses on distinguishing redundant information (features in overlapping areas) and key information (features in non-overlapping areas). The result demonstrates that it has a significant effect on reducing the impact of factors irrelative to classes.

### 4.2. Effectiveness of the Ensemble Convolutional Neural Network

Ensemble learning methods often have better classification performance by combining multiple base classifiers [48]. Li built different ensemble learning models to accurately distinguish 10 different objects’ odors by using different combinations of machine learning classifiers and reported that ensemble learning could significantly improve classification accuracy [49]. Wijaya et al. integrated random forest and AdaBoost to classify beef quality and predict microbial populations in beef through regression models. The ensemble model achieved a higher accuracy than common classifiers such as SVR and random forest [50]. 

Moreover, with the rapid development of deep learning, neural network models have also been introduced into the ensemble learning framework for fault detection, classification, and regression. Wang et al. [51] built an ensemble neural network for joint decision-making of multiple MLP networks and applied it to the rapid detection of alkane gases in electronic noses. Wang et al. [52] designed several ANNs. Each ANN was trained separately with a portion of sensor data, and they finally used the weighed voting strategy to achieve high prediction accuracy.

These studies suggested that a combination of multiple neural networks for ensemble decision-making strategies is effective in achieving an accurate classification. In this study, an attempt was made to obtain base classifiers, which had different abilities of pattern recognition and multi-scale feature extraction, by changing the size and number of convolutional kernels. A CNN model was used to replace the voting process in traditional ensemble learning. The result showed that the ECNN model had better classification results than traditional pattern recognition methods or single base classifiers in the early detection of rotten potatoes.

## 5. Conclusions

In this study, we took the early detection of rotting potatoes as an example and proposed a solution, which consisted of a feature discretization method and an ensemble convolutional neural network, to reduce the influence of ambient gases on odor detection performed by an electronic nose. The main results were as follows: (1) In a storage environment, the prediction accuracies of different classification algorithms for three categories of rotting potatoes were improved by a feature discretization method (SVM: 3.39%, LR: 2.38%, KNN: 8.56%, ECNN: 3.73%). The result proved the feature discretization method can reduce the interference of ambient gases and improve the classification performance of the electronic nose. (2) The ensemble convolutional neural network achieved the highest prediction accuracy in detecting the degree of rot in potatoes (laboratory: 94.70%, storage: 90.76%) compared to traditional pattern recognition methods (SVM: 81.44%, LR: 84.43%, KNN: 79.39%, CNN: 87.02%). This means that the ECNN performs better in terms of feature extraction and effectiveness of decision-making.

The feature discretization method retains differential features and removes redundant feature information by setting category intervals and discretization. On this basis, the ECNN extracts deep features using multiple base classifiers and integrates the results using a convolutional neural network. This method can be used in other detection scenarios using E-noses in the presence of complex ambient gases. The results proved that the ensemble convolutional neural network based on discretized features performed well in the early detection of rotten potatoes. However, there were still some problems that need to be resolved in the future: (1) optimization of temperature control in the gas chamber to reduce the impact of temperature during sampling; (2) suppression of long-term drifts of sensors by using drift correction algorithms; (3) combining miniaturized electronic noses with distributed monitoring modes to adapt to large-scale storage facilities.

In the future, the proposed method may be more universally used in the online quality detection of agricultural products or foods, especially in complex odor environments. Electronic noses can collect data under realistic detection scenarios rather than in the laboratory, utilize feature discretization methods to reduce the impact of ambient gases, and train an ensemble CNN to distinguish between different qualities of agricultural products.

## Figures and Tables

**Table 1:** The response characteristics of sensors used in the E-nose.

Sensor Number	Sensor Name	Main Response Characteristics	Reference
S1	MQ8	Hydrogen	100–1000 ppm
S2	TGS2600	Hydrogen, ethanol, methane, isobutane	1–30 ppm
S3	TGS2602	Ammonia and hydrogen sulfide	1–30 ppm
S4	MQ135	Ammonia, hydrogen sulfide, benzene	10–1000 ppm
S5	TGS2603	Ethanol, trimethylamine, hydrogen sulfide	1–100 ppm
S6	TGS2609	Hydrogen, carbon monoxide	1–30 ppm
S7	MQ136	Hydrogen sulfide	1–200 ppm
S8	TGS2611	Methane	1–500 ppm
S9	TGS2620	Vapors of organic solvents, alcohol, methanol	50–5000 ppm
S10	MQ138	Methylbenzene, acetone, ethanol, methanal	5–500 ppm
S11	TGS2610	Propane, butane	500–5000 ppm
S12	TGS2612	Methane, propane, butane	500–5000 ppm

**Table 2:** The experimental parameters of the E-nose.

Experimental Parameters	Value
pre-heating time	60 min
pre-cleaning time	60 s
injection time	90 s
cleaning time	90 s
sampling frequency	1 Hz
headspace time	10 min
pre-cleaning/cleaning rate	6.5 L/min
sample injection rate	5 L/min

**Table 3:** Feature values of each sensor’s signal.

Feature Properties	Type	Value
Mean value	Time domain	$F_{1}=\frac{1}{T}\sum_{t=1}^{T}x_{t}$
Maximum value	Time domain	$F_{2}=\max(x_{t})$
Area value during injecting	Time domain	$F_{3}=\sum_{t=0}^{T_{b}}x_{t}$
Average stable value	Time domain	$F_{4}=\frac{1}{T_{b}-T_{a}}\sum_{t=T_{a}}^{T_{b}}x_{t}$
Maximum difference value	Time domain	$F_{5}=\max(\frac{\Delta x_{t}}{\Delta t})$
Average difference value	Time domain	$F_{6}=\mathrm{average}(\frac{\Delta x_{t}}{\Delta t})$
Maximum second-order difference	Time domain	$F_{7}=\max(\frac{\Delta^{2}x_{t}}{\Delta t^{2}})$
The biggest five amplitudes after Fast Fourier Transform	Frequency domain	$F_{i}=A(w_{i-7}),i=8,\ldots ,12$

*x_t_* indicates the response value at time *t*; *T* indicates the length of total time; *T_a_* indicates the time when the signal reaches the peak value for the first time; *T_b_* indicates the end of the injection time; *A*, *w* indicate the amplitude value and frequency variable of the frequency spectrum after Fast Fourier Transform.

**Table 4:** Prediction results of different classification methods in the laboratory.

Classification Algorithm	Without Discretization		Discretization		↑
	Training Acc (%)	Test Acc (%)	Training Acc (%)	Test Acc (%)	Acc (%)
SVM	92.42 ± 1.28	**89.17** ± 0.45	93.56 ± 0.83	89.11 ± 0.57	−0.06
LR	90.45 ± 1.28	85.08 ± 0.87	92.23 ± 1.23	**87.05** ± 0.67	1.97
KNN	89.02 ± 1.38	83.94 ± 0.88	95.15 ± 0.89	**86.74** ± 0.74	2.80
Single CNN	94.55 ± 1.06	90.98 ± 0.69	96.74 ± 1.18	**91.67** ± 0.51	1.01
ECNN	98.11 ± 0.78	93.11 ± 0.53	98.86 ± 0.61	**94.70** ± 0.45	1.59

“↑” indicates changes in the mean values of test acc for different classification algorithms after using the feature discretization method. The bold data represent the higher mean values of test accuracy.

**Table 5:** Prediction results of different classification methods in storage.

Classification Algorithm	Without Discretization		Discretization		↑
	Training Acc (%)	Test Acc (%)	Training Acc (%)	Test Acc (%)	Acc (%)
SVM	87.42 ± 0.67	77.95 ± 1.12	89.70 ± 1.10	**81.44** ± 0.36	3.49
LR	90.68 ± 0.58	82.05 ± 0.81	87.73 ± 0.83	**84.43** ± 1.18	2.38
KNN	79.62 ± 0.96	70.83 ± 0.91	83.03 ± 1.25	**79.39** ± 0.77	8.56
Single CNN	89.39 ± 1.23	84.29 ± 0.96	93.64 ± 0.95	**87.02** ± 1.03	2.73
ECNN	93.41 ± 0.67	87.03 ± 0.80	97.95 ± 1.27	**90.76** ± 0.37	3.73

“↑” indicates changes in the mean values of test acc for different classification algorithms after using the feature discretization method. The bold data represent the higher mean values of test accuracy.

## Data Availability

The data presented in this study are available on request from the corresponding author.
